# Assessment of the efficacy of early versus delayed mobility exercise after arthroscopic rotator cuff repair

**DOI:** 10.1007/s00264-025-06477-5

**Published:** 2025-03-07

**Authors:** Hong Tang, Pushan Yang, Xu Wang, Biao Zhao, Kun Ling

**Affiliations:** Orthopedics Department, Guangyuan Central Hospital, Guangyuan, China

**Keywords:** Efficacy, Early mobility exercise, Delayed mobility exercise, Arthroscopic rotator cuff repair

## Abstract

**Purpose:**

Rotator cuff tears were a prevalent cause of shoulder pain and impairment, often necessitating arthroscopic rotator cuff repair. The optimal timing of postoperative mobilization initiation remains a subject of debate implicating patient outcomes. Therefore, this study aimed to evaluate the effectiveness of early and delayed mobilization after arthroscopic rotator cuff repair.

**Methods:**

A total of 84 patients who underwent unilateral arthroscopic rotator cuff repair were included in the study and divided into early and delayed mobility exercise groups. Outcome measures included range of motion, shoulder strength, pain assessment, re-tear rates, return to work and pre-injury activity, as well as patient-reported outcomes at various postoperative time points.

**Results:**

Early mobility exercise after arthroscopic rotator cuff repair led to a significantly greater recovery of range of motion at six weeks postoperatively (*P* < 0.05) and shoulder strength at 12 weeks postoperatively (*P* < 0.05), as compared to the delayed mobility exercise group. However, the early mobility exercise resulted in non-significant excess in the pain assessment at the six-month postoperative mark (*P* > 0.05). Additionally, there were no statistically significant differences between the two groups in several outcome measures, including re-tear rates, return to work and pre-injury activity, and long-term patient-reported outcomes at one year post-operatively (*P* > 0.05).

**Conclusion:**

Both early and delayed mobilization exercises safely improve range of motion, shoulder strength, and pain relief after arthroscopic rotator cuff repair. Early mobilization within six to 12 weeks post-surgery enhances range of motion and strength without increasing re-tear rates.

**Trial registration:**

Not applicable.

## Introduction

Rotator cuff tears were a common source of shoulder pain and disability, affecting a significant portion of the adult population [1, 2, 3]. Arthroscopic rotator cuff repair has become a widely adopted surgical intervention for the management of symptomatic rotator cuff tears, aiming to restore function and alleviate pain in affected individuals [4, 5, 6]. Following surgical intervention, postoperative rehabilitation plays a pivotal role in facilitating patients’ recovery, enhancing range of motion, promoting shoulder strength, and minimizing postoperative complications. Among the key components of postoperative rehabilitation, the timing of mobilization initiation has garnered considerable attention as a potential determinant of functional outcomes and patient recovery following arthroscopic rotator cuff repair.

Numerous studies have explored the optimal timing of mobilization initiation after rotator cuff repair, with divergent findings and recommendations. In the realm of shoulder rehabilitation, the debate between early and delayed initiation of mobilizations remains an area of active investigation. Early mobilization proponents argue that initiating controlled shoulder mobilization and muscle activation exercises soon after surgery may prevent the formation of excessive scar tissue, facilitate joint lubrication, and promote early recovery of range of motion and shoulder strength [7, 8, 9]. Conversely, advocates of delayed mobilization emphasize the need for protective immobilization in the early postoperative period to allow for optimal healing of the repaired tendon and to minimize the risk of re-injury [10, 11, 12, 13]. Despite these divergent viewpoints, there was a paucity of consensus regarding the optimal timing and potential impact of early versus delayed mobilization after arthroscopic rotator cuff repair on patient outcomes and postoperative complications.

To contribute to the growing body of evidence on this topic, the present study aimed to assess the efficacy of early versus delayed mobilization after arthroscopic rotator cuff repair.

## Materials and methods

### Study design

This study was a retrospective cohort analysis that included 84 patients diagnosed with shoulder rotator cuff tears and admitted to our hospital between January 2019 and December 2023. Given that this retrospective study only uses de-identified patient data, there was no risk for harm or impact on patient care. Therefore, informed consent was waived. This waiver was approved by our hospital’s Ethics Review Committee and Ethical Commission, in line with regulatory and ethical guidelines for retrospective studies.

### Eligibility criteria

#### Inclusion criteria

(1) MRI diagnosis of shoulder cuff injury with symptoms of pain or shoulder functional impairment; (2) age ≥ 18 years; (3) first-time unilateral arthroscopic shoulder cuff repair surgery; (4) complete medical records and follow-up of ≥ one year; (5) informed consent to participate in this study.

#### Exclusion criteria

(1) Diagnosis of large tears according to the DeOrio and Cofield shoulder cuff classification; (2) shoulder joint mobility impairment due to central or peripheral nerve injury; (3) upper limb fractures combined with trauma; (4) combined labral injuries; (5) severe peripheral vascular disease or other conditions in the affected limb, (6) precluding rehabilitation exercises.

### Grouping criteria

This study included a total of 84 patients who underwent arthroscopic rotator cuff repair. Patients were divided into early mobilization group 45 people and delayed mobilization group 39 people based on different rehabilitation training methods. Both groups underwent three-phase functional rehabilitation training tailored to the patients’ physical tolerance. The specific details were as follows:

### Early mobilization group

1. Postoperative days zero to five: Abduction exercises and passive stretching of the affected shoulder, with intensity based on patient tolerance, achieving flexion of up to 140°, lateral rotation of the arm to 40°, and external rotation of 60°.
2. Postoperative days six to12: Passive and resistance-free shoulder joint exercises. Upon absence of significant pain, daily living activities were initiated, transitioning flexion to 160°, rotation to 60°, and abduction to 90°, followed by active exercise, focusing strength training on a 90° abduction.
3. After 13 days: Gradual strengthening of resistance training, rotator cuff and deltoid muscle training, active shoulder joint exercises for flexibility, coordination, and stability.

Delayed mobilization group:

1. Postoperative weeks zero to six: Passive shoulder joint mobilization training.
2. Postoperative weeks six to12: Shoulder joint control, proprioception, and muscle strength training.
3. Postoperative weeks 12–20: Full range of motion shoulder joint exercises.

### General information

Demographic data, including age, gender, body mass index (BMI), and smoking history, were collected and documented from the medical records system. Additional information concerning the patients’ dominant hand and baseline pain status was also collected. At six- and twelve-weeks postoperative, the patients’ shoulder joint mobility were recorded, including flexion, abduction, external rotation, internal rotation, and horizontal adduction.

### Pain assessment

At the six-month postoperative mark, both groups of patients underwent pain Assessment, including the Visual analogue scale (VAS) scores, the Constan-Murley Shoulder Score (CMS), the Disabilities of the Arm, Shoulder, and Hand Score (DASH score), Single Assessment Numeric Evaluation (SANE), and Range of Motion.

The VAS pain assessment involved using a 10 cm horizontal line where the patient was informed that the left end represents “no pain” and the right end represents the “most severe pain imaginable.” The patient was then instructed to mark the level of pain currently being experienced on the line. The reliability of the assessment was confirmed with a Cronbach’s alpha value of 0.74 [14].

The CMS evaluation was conducted, encompassing four key aspects: pain, daily life functionality, muscle strength, and range of motion, scoring from 0 to 100, with higher scores indicating better shoulder joint mobility. Additionally, the UCLA score, comprising muscle strength, subjective satisfaction, and active forward flexion angle, was assessed, with scores ranging from 0 to 35 and higher scores indicating improved shoulder joint mobility. The reliability of these assessments was confirmed with a Cronbach’s alpha value of 0.95 [15].

The DASH score [16], designed to measure disability in patients with upper limb disorders, comprises 30 indicators related to daily life activities, with each corresponding to a five-point rating scale ranging from no difficulty (0 points) to unable to perform (5 points). The DASH score was calculated using the formula DASH Score = (Total of the 30 items/30 − 1) * 25, transforming the original score to a scale of 100, where higher scores indicate more severe functional impairment. The reliability of the DASH questionnaire was verified with a Cronbach’s alpha coefficient of 0.938 [17].

Furthermore, the SANE, Patient-Reported Outcomes Measurement Information System-Upper Extremity (PROMIS-UE), and Quick-Disabilities of the Arm, Shoulder, and Hand (Quick DASH) data were routinely captured electronically at our hand and upper extremity center, with a Cronbach’s alpha coefficient of 0.86 [18].

### Re-tear rates and return to work and activity

At the 1-year postoperative follow-up, the patients were examined to determine the occurrence of recurrent rotator cuff tears within the previous year, and the rate of such recurrences was recorded. The patients’ return to work timeframe in weeks and their percentage of return to pre-injury activity were also documented.

### Patient-reported outcomes

At the one year postoperative follow-up, patient self-assessment reports were collected, including the Subjective Shoulder Value (SSV) (0-100), Shoulder Pain and Disability Index (0-100), and Simple Shoulder Test (SST) [19] (0–12). The SST serves as a brief patient-reported outcome measure, evaluating functional limitations of the affected shoulder in individuals with shoulder impairment. Its reliability was confirmed with a Cronbach’s alpha of 0.78 [20]. The SSV represents a patient’s subjective evaluation of the shoulder, expressed as a percentage of a completely healthy shoulder, which would score 100%. Its reliability, indicated by a Cronbach’s alpha of above 0.70, was also verified [21].

### Statistical analysis

The data were analyzed using SPSS 29.0 statistical software (SPSS Inc, Chicago, IL, USA). Categorical data were presented as [n (%)] format. For sample sizes ≥ 40 and theoretical frequency T ≥ 5, the chi-square test was conducted using the basic formula. When the sample size was ≥ 40 but the theoretical frequency was 1 ≤ T<5, the chi-square test was performed using the corrected formula. For sample sizes < 40 or theoretical frequency T < 1, statistical analysis was carried out using Fisher’s exact probability method. The normality of continuous variables was assessed using the Shapiro-Wilk test. Continuous variables with normal distribution were expressed as (‾χ ± s) and analyzed using the t-test with corrected variance. Non-normally distributed data were presented in the form of median (25th percentile, 75th percentile) and analyzed using the Wilcoxon rank-sum test. A two-tailed *P*-value < 0.05 was considered statistically significant.

## Results

### Patient demographics

In this study evaluating the efficacy of early versus delayed mobility exercise after arthroscopic rotator cuff repair, a total of 84 patients were included, with 45 patients were assigned to the early mobility exercise group and 39 patients were assigned to the delayed mobility exercise group (Table 1). The mean age was 45.81 years in the early mobility exercise group and 46.73 years in the delayed mobility exercise group, with no statistically significant difference between the groups (*P*>0.05). Similarly, no significant differences were found between the groups in terms of gender distribution, BMI, smoking history, dominant arm, or baseline pain scores (*P*>0.05). These results suggest a well-matched distribution of demographic and baseline characteristics between the two groups, reinforcing the comparability of the two intervention arms.

**Table 1:** Patient demographics

Parameter	Early Mobility Exercise (*n* = 45)	Delayed Mobility Exercise (*n* = 39)	t	*P*
Age (years)	45.81 ± 4.67	46.73 ± 5.42	0.826	0.411
Gender (M/F)	24 (53.33%) / 21 (46.67%)	21 (53.85%) / 18 (46.15%)	0.000	1.000
BMI (kg/m^2^)	25.74 ± 2.61	26.05 ± 2.19	0.597	0.552
Smoking history (Y/N)	13 (28.87%) / 32 (71.13%)	11 (28.21%) / 28 (71.79%)	0.000	1.000
Dominant arm (R/L)	43 (95.56%) / 2 (4.44%)	37 (94.87%) / 2 (5.13%)	0.000	1.000
Baseline pain (0–10)	7.45 ± 1.12	7.21 ± 1.08	0.979	0.331

### Range of motion at six weeks post-op

**Fig. 1:**
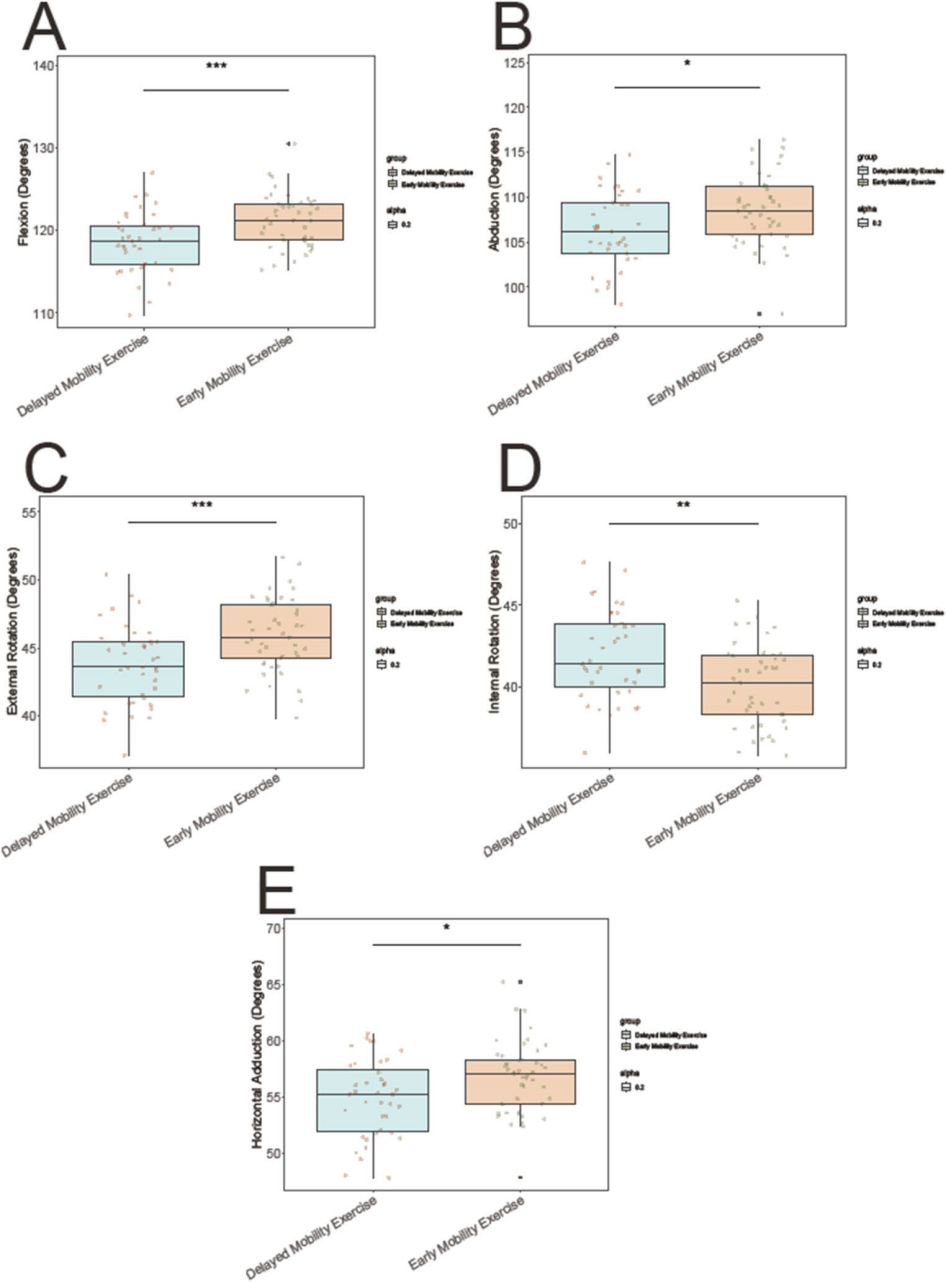
The early mobility exercise group vs. the delayed mobility exercise group. Range of Motion at 6 Weeks Post-Op including **(A)** Flexion, **(B)** Abduction, **(C)** External rotation, **(D)** Internal rotation, and **(E)** Horizontal adduction

The early mobility exercise group demonstrated statistically significant improvements in range of motion at six Weeks Post-Op compared to the delayed mobility exercise group. Specifically, the early mobility group exhibited greater flexion (121.14 ± 3.21 vs. 118.46 ± 3.67 degrees; t = 3.54, *P* < 0.001), abduction (108.52 ± 3.87 vs. 106.45 ± 4.12 degrees; t = 2.595, *P* = 0.011), external rotation (45.93 ± 2.67 vs. 43.78 ± 2.89 degrees; t = 3.526, *P* < 0.001), internal rotation (40.06 ± 2.45 vs. 41.95 ± 2.71 degrees; t = 3.331, *P* = 0.001), and horizontal adduction (56.34 ± 3.23 vs. 54.89 ± 3.45 degrees; t = 2.522, *P* = 0.014) (Fig. 1). These results suggest that early mobilization following arthroscopic rotator cuff repair may confer significant benefits in improving post-operative range of motion outcomes.

### Shoulder strength at 12 weeks post-op

**Fig. 2:**
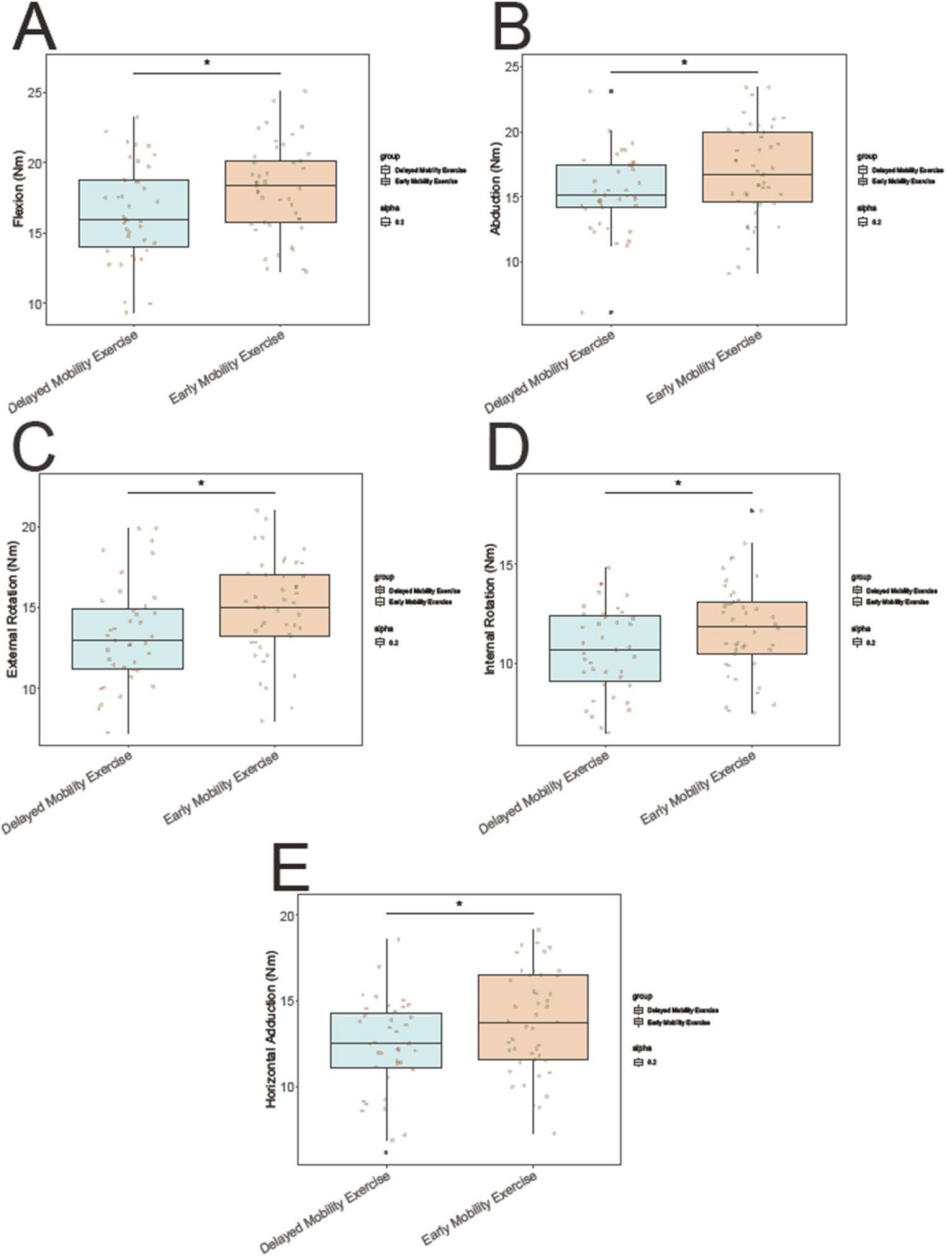
The early mobility exercise group vs. the delayed mobility exercise group. Shoulder Strength at 12 Weeks Post-Op including **(A)** Flexion, **(B)** Abduction, **(C)** External rotation, **(D)** Internal rotation, and **(E)** Horizontal adduction

The early mobility exercise group demonstrated significantly greater shoulder strength at 12 Weeks Post-O compared to the delayed mobility exercise group. Specifically, the early mobility exercise group demonstrated higher shoulder strength in flexion (18.12 ± 3.21 vs. 16.48 ± 3.45 Nm; t = 2.248, *P* = 0.027), abduction (16.85 ± 3.56 vs. 15.32 ± 2.98 Nm; t = 2.147, *P* = 0.035), external rotation (14.97 ± 2.98 vs. 13.36 ± 3.12 Nm; t = 2.398, *P* = 0.019), internal rotation (11.78 ± 2.34 vs. 10.67 ± 2.21 Nm; t = 2.247, *P* = 0.027), and horizontal adduction (13.77 ± 2.98 vs. 12.31 ± 2.76 Nm; t = 2.317, *P* = 0.023) (Fig. 2). These findings highlight the potential benefits of early mobility exercise in enhancing shoulder strength following arthroscopic rotator cuff repair.

### Pain assessment at six months post-op

The assessment of pain at six months post-operatively in patients undergoing early versus delayed mobility exercise following arthroscopic rotator cuff repair revealed no statistically significant differences between the two groups in several outcome measures. Specifically, the VAS (2.32 ± 0.98 vs. 2.45 ± 1.07; t = 0.595 *P* = 0.554), CMS (91.27 ± 3.21 vs. 90.68 ± 3.98; t = 0.747, *P* = 0.458), DASH (5.43 ± 1.23 vs. 5.67 ± 1.34; t = 0.846, *P* = 0.4), Single Assessment Numeric Evaluation (9.78 ± 0.87 vs. 9.65 ± 0.98; t = 0.608, *P* = 0.545), and Range of Motion (177.34 ± 3.67 vs. 176.45 ± 3.89 degrees; t = 1.073, *P* = 0.287) (Table 2). These findings suggest that, at 6 months post-operatively, pain levels and functional outcomes were comparable between the early and delayed mobility exercise groups, indicating that the timing of mobility exercises may not significantly influence these outcomes at this stage.

**Table 2:** Pain assessment at 6 months Post-Op of the early mobility exercise group vs. the delayed mobility exercise group

Parameter	Early Mobility Exercise (*n* = 45)	Delayed Mobility Exercise (*n* = 39)	t	*P*
VAS (0–10)	2.32 ± 0.98	2.45 ± 1.07	0.595	0.554
CMS	91.27 ± 3.21	90.68 ± 3.98	0.747	0.458
DASH	5.43 ± 1.23	5.67 ± 1.34	0.846	0.4
SANE	9.78 ± 0.87	9.65 ± 0.98	0.608	0.545
Range of Motion (Degrees)	177.34 ± 3.67	176.45 ± 3.89	1.073	0.287

### Re-tear rates at one year post-op

**Fig. 3:**
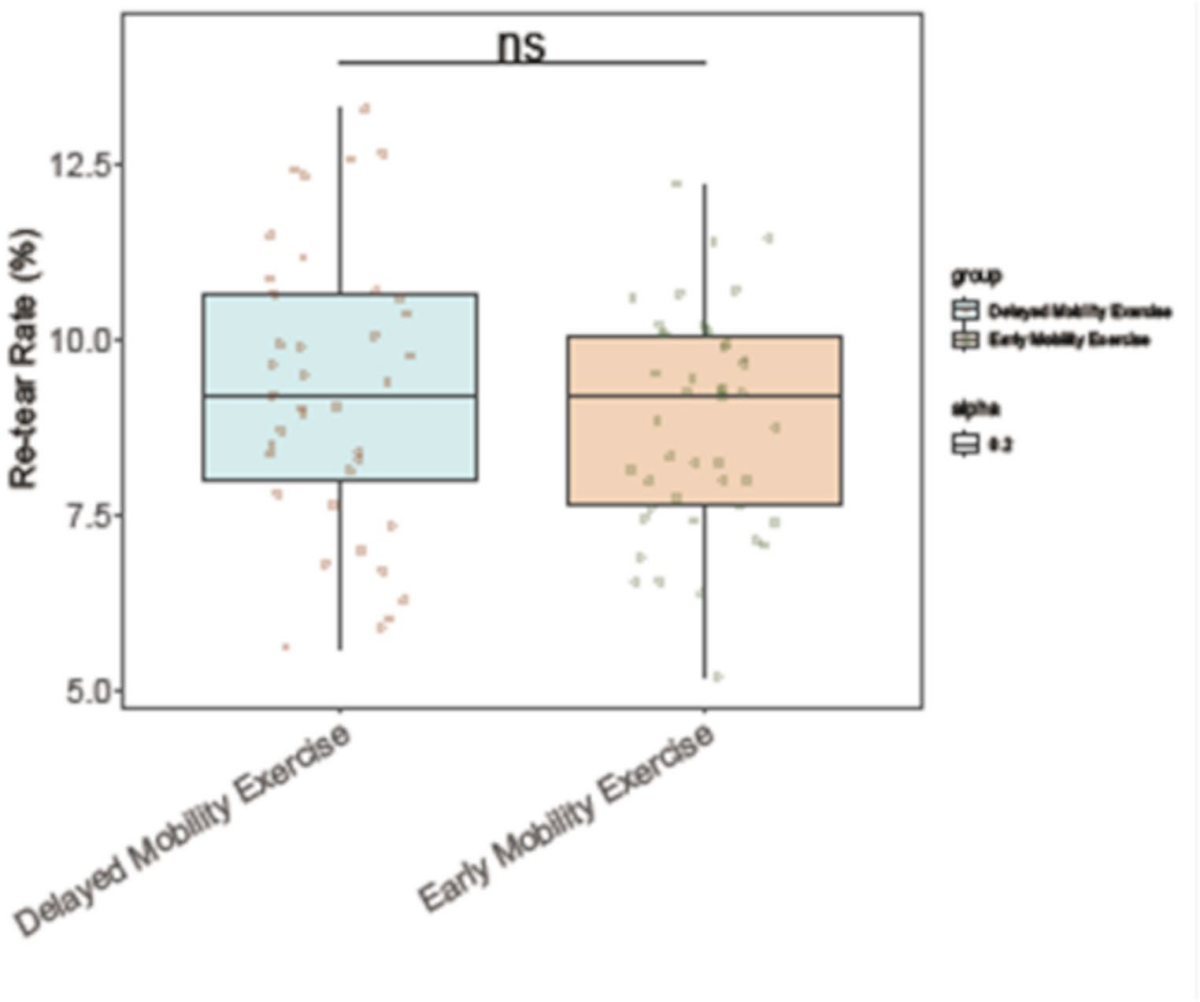
The early mobility exercise group vs. the delayed mobility exercise group. Re-tear Rates at 1 Year Post-Op

The comparison of re-tear rates at one year post-operatively between patients undergoing early versus delayed mobility exercise following arthroscopic rotator cuff repair showed no statistically significant difference. The re-tear rates of 8.83% ± 1.54% in the early mobility exercise group and 9.25% ± 2.01% in the delayed mobility exercise group (t = 1.049, *P* = 0.298) (Fig. 3). These results suggest that the timing of initiating mobility exercises may not significantly affect the incidence of re-tears at the one year post-operative follow-up.

### Return to work and activity at one year post-op

The comparison of return to work and pre-injury activity between the early and delayed mobility exercise groups after arthroscopic rotator cuff repair revealed no statistically significant differences (Table 3). Specifically, the early mobility exercise group demonstrated a return to work of 18.76 ± 2.98 weeks compared to 19.89 ± 3.21 weeks in the delayed mobility exercise group (t = 1.667, *P* = 0.1), and a return to pre-injury activity of 91.34% ± 2.45% compared to 90.67% ± 2.78% in the delayed mobility exercise group (t = 1.174, *P* = 0.244). These findings suggest that the timing of mobility exercise initiation may not significantly impact the return to work and pre-injury activity measures at the assessed time points.

**Table 3:** Return to work and activity of the early mobility exercise group vs. the delayed mobility exercise group

Parameter	Early Mobility Exercise (*n* = 45)	Delayed Mobility Exercise (*n* = 39)	t	*P*
Return to Work (weeks)	18.76 ± 2.98	19.89 ± 3.21	1.667	0.1
Return to Pre-injury Activity (%)	91.34 ± 2.45	90.67 ± 2.78	1.174	0.244

### Patient-reported outcomes at one year post-op

The patient-reported outcomes at one year post-operatively between the early and delayed mobility exercise groups after arthroscopic rotator cuff repair showed no statistically significant differences (Table 4). Specifically, the SSV was 88.49 ± 4.32 in the early mobility exercise group compared to 87.73 ± 4.56 in the delayed mobility exercise group (t = 0.78, *P* = 0.438), the shoulder pain and disability index was 6.78 ± 1.54 compared to 6.89 ± 1.67 (t = 0.325, *P* = 0.746), and the SST was 10.78 ± 0.98 compared to 10.43 ± 1.12 (t = 1.517, *P* = 0.133). These findings suggest that the timing of mobility exercise initiation may not significantly impact the patient-reported outcomes at the 1-year post-operative time point.

**Table 4:** Patient-reported outcomes at 1 year Post-Op of the early mobility exercise group vs. the delayed mobility exercise group

Parameter	Early Mobility Exercise (*n* = 45)	Delayed Mobility Exercise (*n* = 39)	t	*P*
Subjective Shoulder Value (0-100)	88.49 ± 4.32	87.73 ± 4.56	0.78	0.438
Shoulder Pain and Disability Index (0-100)	6.78 ± 1.54	6.89 ± 1.67	0.325	0.746
Simple Shoulder Test (0–12)	10.78 ± 0.98	10.43 ± 1.12	1.517	0.133

## Discussion

Rotator cuff tears are the most common upper extremity condition seen by primary care and orthopaedic surgeons, with a spectrum ranging from tendinopathy to full-thickness tears with arthritic change [22, 23, 24]. Arthroscopic rotator cuff repair has become the gold standard management for rotator cuff repair [25, 26, 27]. The present study aimed to assess the efficacy of early versus delayed mobility exercise after arthroscopic rotator cuff repair. The study revealed that patients undergoing early mobility exercise exhibited superior range of motion at six weeks post-operatively compared to those in the delayed mobility exercise group. This finding was consistent with previous research suggesting that early mobilization may lead to improved early postoperative outcomes in terms of range of motion. The potential mechanisms behind this superiority could be related to the prevention of excessive scar tissue formation and joint stiffness through early mobilization, leading to improved flexibility and functional recovery [28, 29]. After surgery, the body initiates a healing response that involves the formation of scar tissue at the site of the repair. Early mobilization may help prevent the adhesion of surrounding tissues, which can limit joint mobility and range of motion. By engaging in early mobility exercises, patients may facilitate the alignment and organization of collagen ffibres during the healing process, leading to improved tissue flexibility and reduced scar tissue formation [30, 31].

Moreover, the early mobility group demonstrated significantly higher shoulder strength at 12 weeks post-operatively. This result may be attributed to the early engagement of muscle activation and strengthening exercises, facilitating better recovery of muscle function and strength in the early mobility exercise group. Synovial fluid acts as a lubricant and provides nourishment to the cartilage and other joint structures. By engaging in early mobility exercises, patients can stimulate the production and circulation of synovial fluid within the joint, which can contribute to enhanced joint lubrication, reduced friction, and improved range of motion [32, 33]. It was worth noting that achieving superior range of motion and strength at early time points postoperatively may potentially contribute to better long-term functional outcomes and patient satisfaction.

Contrary to the findings on range of motion and strength, the assessment of pain at six months postoperatively did not reveal significant differences between the early and delayed mobility exercise groups. This result suggests that the timing of mobility exercise initiation may not have a substantial impact on pain levels at the six month postoperative time point. The lack of significant difference in pain assessment levels between the groups might be influenced by various factors, including patient-specific pain thresholds, individual pain management strategies, and the multifactorial nature of pain perception. Additionally, the management of postoperative pain might have been standardized across both groups, potentially mitigating the impact of mobility exercise timing on pain outcomes.

Re-tear rates at one year postoperatively did not differ significantly between the early and delayed mobility exercise groups. This finding indicates that the timing of mobility exercise initiation might not be a significant factor influencing the incidence of re-tears following arthroscopic rotator cuff repair. The comparable re-tear rates between the groups could be attributed to the effectiveness of the surgical repair and postoperative care protocols independent of the timing of mobility exercise initiation. Furthermore, return to work and pre-injury activity measures also did not show statistically significant differences between the groups. This suggests that the timing of mobility exercise initiation may not significantly impact the return to work and pre-injury activity.

Moreover, no significant differences were observed in patient-reported outcomes at the one year postoperative time point between the early and delayed mobility exercise groups. This finding indicates that the timing of mobility exercise initiation may not significantly impact long-term patient-reported outcomes, including SSV, shoulder pain and disability index, and SST scores. The uniformity in long-term patient-reported outcomes between the groups may be indicative of the comprehensive rehabilitation and postoperative care provided to all patients, mitigating the potential impact of the timing of mobility exercise initiation on these outcomes.

It was important to consider several limitations of the study when interpreting these findings. The retrospective nature of the study introduces inherent biases and limitations, including potential selection bias, the reliance on existing medical records, and the inability to control for all confounding variables. Additionally, the sample size of the study may have influenced the statistical power to detect significant differences in certain outcomes. Furthermore, the study’s follow-up duration may be insufficient to capture potential long-term differences in outcomes between the early and delayed mobility exercise groups.

## Conclusion

In conclusion, the findings of this study support the potential benefits of early postoperative mobility exercise following arthroscopic rotator cuff repair in terms of range of motion and shoulder strength at early time points postoperatively. However, the timing of mobility exercise initiation may not significantly impact pain levels, re-tear rates, return to work and pre-injury activity, and long-term patient-reported outcomes. Future prospective studies with larger sample sizes and longer follow-up durations were warranted to further elucidate the optimal timing and specific protocols for postoperative mobility exercise after arthroscopic rotator cuff repair.

## Data Availability

The dataset generated or analyzed in this study can be provided under reasonable request from corresponding author (Kun Ling, 18113687027@163.com).
